# Cops2 promotes pluripotency maintenance by Stabilizing Nanog Protein and Repressing Transcription

**DOI:** 10.1038/srep26804

**Published:** 2016-05-26

**Authors:** Weiyu Zhang, Peiling Ni, Chunlin Mou, Yanqin Zhang, Hongchao Guo, Tong Zhao, Yuin-Han Loh, Lingyi Chen

**Affiliations:** 1 State Key Laboratory of Medicinal Chemical Biology, Collaborative Innovation Center for Biotherapy, 2011 Collaborative Innovation Center of Tianjin for Medical Epigenetics, Tianjin Key Laboratory of Protein Sciences and College of Life Sciences, Nankai University, Tianjin 300071, China; 2 Epigenetics and Cell Fates Laboratory, A*STAR Institute of Molecular and Cell Biology, 61 Biopolis Drive Proteos, Singapore 138673, Singapore; 3 Department of Biological Sciences, National University of Singapore, Singapore; 4 State Key Laboratory of Molecular Oncology, Cancer Institute/Hospital, Chinese Academy of Medical Sciences, Beijing 100021, China

## Abstract

The COP9 signalosome has been implicated in pluripotency maintenance of human embryonic stem cells. Yet, the mechanism for the COP9 signalosome to regulate pluripotency remains elusive. Through knocking down individual COP9 subunits, we demonstrate that Cops2, but not the whole COP9 signalosome, is essential for pluripotency maintenance in mouse embryonic stem cells. Down-regulation of Cops2 leads to reduced expression of pluripotency genes, slower proliferation rate, G2/M cell cycle arrest, and compromised embryoid differentiation of embryonic stem cells. Cops2 also facilitates somatic cell reprogramming. We further show that Cops2 binds to Nanog protein and prevent the degradation of Nanog by proteasome. Moreover, Cops2 functions as transcriptional corepressor to facilitate pluripotency maintenance. Altogether, our data reveal the essential role and novel mechanisms of Cops2 in pluripotency maintenance.

Embryonic stem cells (ESCs) are able to self-renew indefinitely, and have the potential to differentiate into all types of cells in the adult organism. The unique property of ESCs, namely pluripotency, renders ESCs as a promising cell source in regenerative medicine and cell replacement therapy. Transcriptional regulation plays a critical role in pluripotency maintenance of ESCs^1^,^2^. Previous studies found that three transcription factors Oct4, Nanog and Sox2 regulate each other and form a core transcriptional regulatory circuitry underlying pluripotency maintenance^3^. This core regulatory circuitry not only activates the expression of pluripotency-associated genes, but also suppresses the expression of differentiation-related genes^3^. Meanwhile, many other pluripotency associated transcription factors and coactivators, including Klf4, Sall4, Esrrb and Ncoa3, regulate the three genes of the core regulatory circuitry, forming an expanded pluripotency regulatory network and allowing signaling pathways integrated into transcriptional regulation^4^,^5^,^6^,^7^,^8^. In addition, the core components of the pluripotency network are regulated at protein level through post-translational modifications. For example, phosphorylation of Ser/Thr-Pro motifs of Nanog promotes the interaction between Nanog and Pin1, and stabilizes Nanog protein^9^. Both Oct4 and Sox2 are modified with O-linked-N-acetylglucosamine (O-GlcNAc). O-GlcNAcylation of Thr 228 enhances the transcriptional activity of Oct4, and regulates the functions of Oct4 in maintaining ESC self-renewal and reprogramming somatic cells^10^.

The COP9 signalosome (CSN), composed of 8 subunits (Cops1 to Cops8), is highly conserved from yeast to human^11^,^12^,^13^. The most studied CSN function is to regulate protein degradation. The CSN suppresses the activity of the cullin-RING-E3 ligases (CRL) through deneddylation of cullins, thus enhancing protein stability^14^,^15^. It also regulates the ubiquitin ligase COP1, consequently the degradation of COP1 substrates^16^. Moreover, CSN-associated deubiquitinating enzymes, Ubp12 in yeast and USP15 in mammals, may stabilize the adaptor subunits of CRL and IκBα, respectively, through deubiquitination^17^,^18^. In addition to regulation of protein degradation, the CSN is also involved in transcriptional regulation, protein phosphorylation and subcellular distribution^19^,^20^,^21^,^22^,^23^. While the CSN functions as a complex, CSN subunits may also have their own functions independent of the CSN complex. For example, Alien, a variant of Cops2, has been demonstrated to be a transcriptional corepressor^24^,^25^.

Through a whole-genome RNAi screening experiment, it has been shown that down-regulation of CSN subunits, COPS1, COPS2 and COPS4, reduces the expression of the *OCT4-GFP* reporter in human ESCs, indicating a role of the CSN in pluripotency maintenance^26^. Consistent with this observation, some CSN subunits are required for mouse embryo development. Homozygous knockout of *Cops2*, *Cops3*, *Cops5*, *Cops6*, or *Cops8* in mice, leads to early embryo death^27^,^28^,^29^,^30^,^31^. No ESCs could be derived from *Cops2* or *Cops8* null blastocysts, implying that the CSN is involved in pluripotency establishment^29^,^30^. Yet, *Cops2*, *Cops3*, *Cops5*, *Cops6*, or *Cops8* knockout embryos die at different embryonic days ranging from day 6.5 to 8.5, implying that individual CSN subunits have their own biological functions, in addition to the function of the CSN. It is not clear whether the whole CSN complex or individual CSN subunits are required for pluripotency maintenance, and how the CSN or individual CSN subunits contribute to pluripotency maintenance.

To elucidate the function and mechanisms of the CSN in pluripotency maintenance, we knocked down individual CSN subunits in mouse ESCs, and found that only Cops2 is essential for pluripotency maintenance in mouse ESCs. We further demonstrated that Cops2 stabilizes Nanog protein through direct interaction. In addition, Cops2 functions as a transcriptional corepressor to suppress gene expression, including 2-cell-stage embryo specific (2C) genes. In summary, our data revealed that Cops2, but not the CSN, is required for pluripotency maintenance in mouse ESCs.

## Results

**Figure 1:**
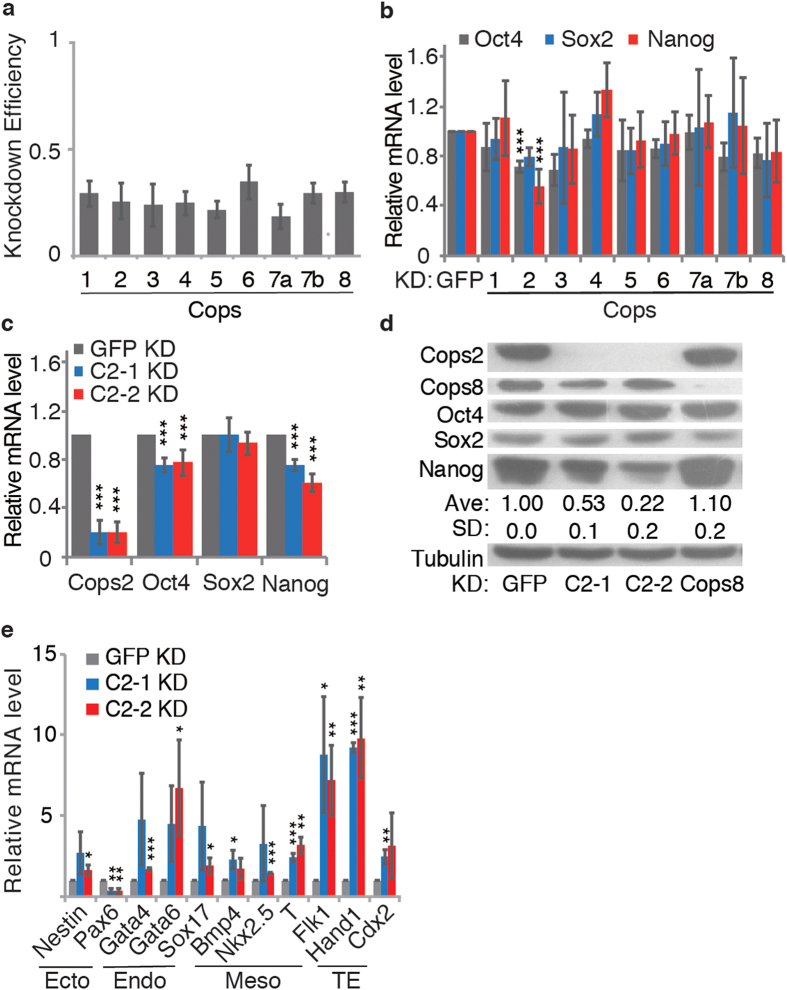
KD of *Cops2*, but not any other CSN subunits, reduces the expression of *Nanog* and *Oct4*. (**a**) The KD efficiencies of shRNAs targeting CSN subunits. Mouse ESCs were transfected with shRNA plasmids targeting *GFP* or CSN subunits. Forty-eight hours later, cells were harvested for RNA prep and quantitative RT-PCR. (**b**) The effect on pluripotency gene expression after KD of CSN subunits. The samples were prepared as described in (**a**). Quantitative RT-PCR was performed to measure the RNA levels of *Nanog*, *Oct4* and *Sox2*. (**c**,**d**) Two shRNAs targeting *Cops2* validate the regulatory effect on *Nanog* and *Oct4*. Mouse ESCs were transfected with plasmids expressing shRNAs targeting different sequences of *Cops2* (C2-1 and C2-2), *GFP* or *Cops8* (C8). Seventy-two hours later, cells were harvested for quantitative RT-PCR (**c**) and Western blot (**d**). The averages (Ave) and standard deviations (SD) of quantified Nanog expression levels from three independent experiments are shown below the blot. (**e**) *Cops2* KD activates the expression of differentiation genes, except for *Pax6*. Quantitative RT-PCR was performed with the same cells described in (**c**). Data are shown as mean ± SD (n = 3).

To clarify the role of CSN subunits in pluripotency maintenance, we examined the expression of pluripotency genes, *Nanog*, *Oct4*, and *Sox2*, upon knocking down each CSN subunit by shRNA in mouse ESCs (Fig. 1a). In contrast to the RNAi screening data in human ESCs^26^, only knockdown (KD) of *Cosp2*, but not any other CSN subunits, reduces the expression of *Nanog* and *Oct4* mRNA (Fig. 1b). To rule out the possibility of shRNA off-target effect, the regulatory effect of Cops2 on *Nanog* and *Oct4* at both RNA and protein levels was further validated with another shRNA targeting *Cops2* (Fig. 1c,d). In addition, differentiation markers of three germ layers and the trophectoderm (TE), except for the ectodermal marker *Pax6*, are up-regulated upon *Cops2* KD in ESCs (Fig. 1e), implicating compromised pluripotent status in *Cops2* KD ESCs.

To further characterize the role of Cops2 in ESCs, we attempted to establish stable *Cops2* KD ESCs. ESCs were transfected with plasmids expressing shRNAs targeting *GFP* and CSN subunits. Much less *Cops2* KD colonies (less than 10 colonies per experiment) grew up after 7–10 day puromycin selection, compared to other CSN subunits and *GFP* KD ESCs (ranging from 50 to 280 colonies) (Supplementary Table S1). Moreover, *Cops2* is inefficiently knocked down in the surviving *Cops2* KD ESC clones (Supplementary Fig. S1). These data imply that Cops2 is essential for the self-renewal of ESCs.

**Figure 2:**
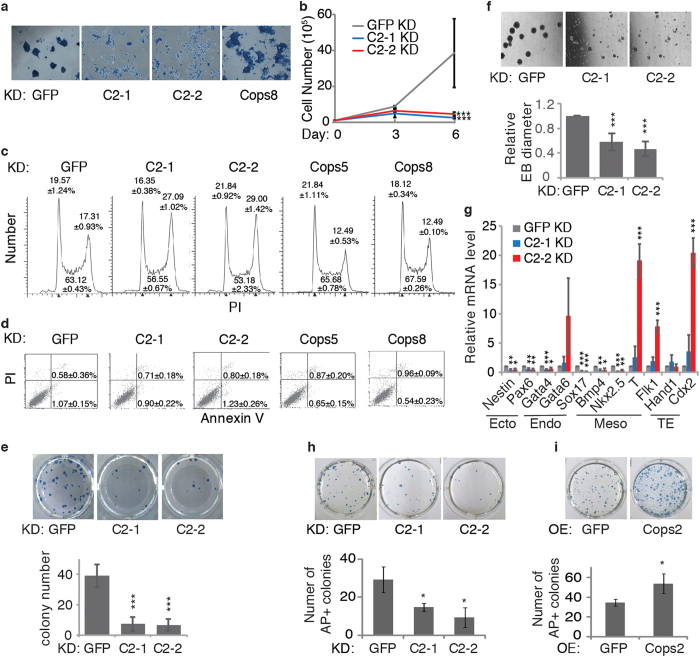
Cops2 is involved in pluripotency maintenance and somatic reprogramming. (**a**) Mouse ESCs were transfected with shRNA plasmids targeting *GFP*, *Cops2* (C2-1 and C2-2) or *Cops8*. Seventy-two hours after transfection, cells were subjected to AP staining. (**b**) At 72 hours after transfection (counted as day 0), *GFP* or *Cops2* KD ESCs were plated 1 × 10^5^ cells/6-well. Then cells were passaged at 1:10 ratio every 3 days. The number of cells was counted on day 3 and day 6. (**c**) and (**d**) Cell cycle (**c**) and apoptosis (**d**) analyses of ESCs after knocking down *GFP*, *Cops2* (C2-1 and C2-2), *Cops5*, and *Cops8* for 3 days. (**e**) Colony forming assay of ESCs with *GFP* or *Cops2* KD. Quantification of data from three independent experiments was plotted. (**f**) EB differentiation of *GFP* or *Cops2* KD ESCs. ESCs were transfected with shRNA plasmids targeting *GFP* or *Cops2*. Three days after transfection, cells were collected for EB differentiation for 4 days. Top panel shows the images of day 4 EBs, and quantified EB diameters were plotted in bottom panel. (**g**) Quantitative RT-PCR analysis of differentiation genes in day 4 EBs, as described in (**f**). (**h**,**i**) Cops2 facilitates somatic cell reprogramming. MEFs were infected with retroviruses expressing shRNAs targeting *GFP* or *Cops2* (**h**), or retroviruses expressing *GFP* or *Cops2* (**i**), together with *Oct4*, *Sox2*, *Klf4*, and c-*Myc* expressing retroviruses. Fourteen days after the initial infection, cells were stained for AP, and AP positive colonies were counted. Top panel shows representative images of reprogrammed cells, and bottom panel shows the quantification results of three independent experiments. Data are shown as mean ± SD (n = 3).

We then switched to knock down *Cops2* transiently in ESCs. KD of *Cops2* impairs the alkaline phosphatase (AP) positivity in ESCs, while *Cops8* KD does not affect the expression of AP (Fig. 2a). In addition, *Cops2* KD slows down the proliferation of ESCs, likely due to G2/M arrest, but not cell apoptosis (Fig. 2b–d). Consistent with the slower proliferation rate of *Cops2* KD ESCs, the colony forming ability of ESCs is also reduced upon *Cops2* KD (Fig. 2e). All these data indicate that KD of *Cops2* compromises the self-renewal of ESCs.

Next, we sought to characterize whether *Cops2* KD affects the differentiation potential of ESCs. Embryoid bodies (EBs) formed by *Cops2* KD ESCs are smaller than control *GFP* KD EBs (Fig. 2f). Moreover, most of the differentiation markers are not fully activated in *Cops2* KD EBs, particularly the ectodermal markers *Nestin* and *Pax6* (Fig. 2g), implying that the differentiation potential of ESCs is impaired upon *Cops2* KD.

To address whether Cops2 plays a role in somatic cell reprogramming, *Cops2* was knocked down or overexpressed in mouse embryonic fibroblasts (MEFs), which were co-infected with retroviruses expressing Oct4, Sox2, Klf4 and c-Myc. *Cops2* KD reduces the reprogramming efficiency, while overexpression of *Cops2* increases the number of AP positive colonies after reprogramming (Fig. 2h,i), implicating that Cops2 facilitates Yamanaka factors to reprogram somatic cells.

**Figure 3:**
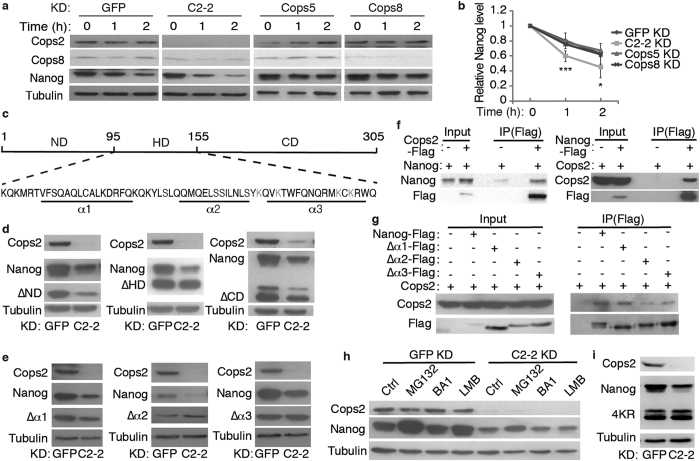
Cops2 binds to and stabilize Nanog protein. (**a**) Three days after transfection, *GFP*, *Cops2*, *Cops5*, and *Cops8* KD cells were treated with cycloheximide for indicated time. Nanog protein levels were measured by Western blot. (**b**) Quantification results of (**a**). Data are shown as mean ± SD (n = 3). (**c**) Schematic illustration of Nanog protein. ND: N-terminal domain; HD: Homeobox domain; CD: C-terminal domain. Four lysine residues in the α3 of the HD are shown in green. (**d**) *Cops2* was knocked down in ESCs expressing Flag-tagged ΔND, ΔHD, or ΔCD Nanog mutants. The expression levels of endogenous Nanog and exogenous mutants were measured by Western blot. ΔND Nanog mutant was detected by Flag antibody. (**e**) Similar to (**d**), except that ESCs expressing Flag-tagged Δα1, Δα2, or Δα3 Nanog mutants were used. (**f**) HEK293T cells were transfected with plasmids expressing Cops2 or Nanog, as indicated. Two days after transfection, Co-IP and Western blot were performed. (**g**) Co-IP experiments to detect the interactions of Cops2 with Flag-tagged WT, Δα1, Δα2, and Δα3 Nanog. (**h**) Three days after transfection, *GFP* and Cops2 KD ESCs were treated with 100 nM bafilomycin A1 (BA), 10 μM MG132, or 60 ng/ml leptomycin B (LMB) for 2 hours, and then subjected to Western blot. (**i**) *Cops2* was knocked down in ESCs expressing 4KR ΔCD Nanog mutant, in which four lysine residues (K138, K141, K150 and K152) in α3 were replaced by arginine. Western blot was performed to measure the expression of endogenous Nanog and exogenous 4KR ΔCD Nanog.

It is noticeable that the down-regulation of Nanog is more eminent at protein level than at RNA level (Fig. 1c,d), implying that Cops2 might regulate Nanog protein stability. To understand how Cops2 regulates the expression of *Nanog*, through transcriptional regulation or by affecting Nanog protein stability, we first performed luciferase reporter assay using a 6-kb *Nanog* promoter. KD of *Cops2* does not affect the *Nanog* promoter activity, whereas knocking down a known *Nanog* activator Esrrb reduces the reporter activity (Supplementary Fig. S2a). It indicates that Cops2 does not regulate the transcription of *Nanog*. Next, we measured the degradation rate of Nanog protein after inhibition of protein synthesis by cycloheximide. Nanog protein is degraded faster upon *Cops2* KD, compared to *GFP*, *Cops5* and *Cops8* KD samples (Fig. 3a,b). Nanog protein contains N-terminal (ND), Homeobox (HD), and C-terminal (CD) domains (Fig. 3c). *Cops2* KD does not affect the expression level of ΔHD mutant, while ΔND and ΔCD mutants are down-regulated upon *Cops2* KD (Fig. 3d). In addition, the activities of HD-luciferase and full-length Nanog-luciferase, but not ND- or CD-luciferase is reduced when *Cops2* is knocked down (Supplementary Fig. S2b). These data suggest that Cops2 regulates Nanog stability through the HD. The HD is composed of three α-helixes, α1, α2 and α3 (Fig. 3c). Deletions of individual α-helixes revealed that α2 and α3, but not α1, are required for Cops2 to regulate Nanog protein stability (Fig. 3e). To distinguish whether Cops2 regulates Nanog stability through direct or indirect interaction, we performed co-immunoprecipitation (co-IP) experiments, and demonstrated that Cops2 and Nanog interact with each other (Fig. 3f). More importantly, only Cops2, but not Cops5, is associated with Nanog (Supplementary Fig. S2c), further suggesting a role of Cops2 independent of the CSN. Consistent with the roles of α2 and α3 in regulating Nanog stability by Cops2, deletion of α2 or α3 impairs the interaction between Nanog and Cops2 (Fig. 3g).

To explore how Nanog is degraded upon *Cops2* KD, *GFP* or *Cops2* KD ESCs were treated with the autophagy inhibitor bafilomycin A1 (BA), the proteasome inhibitor MG132, or the nuclear export inhibitor leptomycin B (LMB). Only MG132 treatment increases the expression level of Nanog (Fig. 3h), regardless of whether *Cops2* is knocked down or not, suggesting that Nanog is mainly degraded by proteasome. Furthermore, when the four lysine residues in the α3 region (Fig. 3c) were replaced with arginine (4KR), the expression level of the 4KR ΔCD mutant remains stable after *Cops2* KD (Fig. 3i), while the interaction between the 4KR ΔCD mutant and Cops2 are unaffected (Supplementary Fig. S2d), implying that these four lysine residues might be potential ubiquitination sites. In addition, we also tested whether Cops2 regulates Nanog protein stability through controlling the translocation of Nanog to the nucleus. However, *Cops2* KD does not alter the cytoplasmic and nuclear distribution of Nanog (Supplementary Fig. S2e), thus ruling out this possibility.

**Figure 4:**
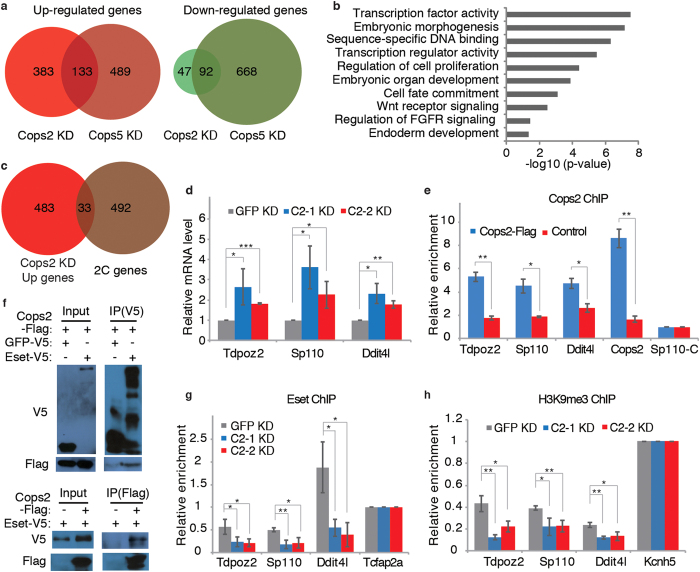
Cops2 acts as a transcriptional corepressor in ESCs. (**a**) Venn diagrams of differentially expressed genes in *Cops2* and *Cops5* KD ESCs, compared to control *GFP* KD ESCs. (**b**) Gene ontology analysis of Cops2 regulated genes. (**c**) Venn diagram shows the overlap between Cops2 repressed genes and 2C genes. *p* = 5.466e-5; Fisher’s exact test. (**d**) The expression of 2C genes, *Tdpoz2*, *Sp110*, and *Ddit4l*, was measured by quantitative RT-PCR in *GFP* and *Cops2* KD ESCs. (**e**) ChIP assays were performed with ESCs expressing Flag-tagged Cops2 and control ESCs. Sp110-C is located at the 3′-end of the *Sp110* gene, and served as a negative control region for Cops2 binding. (**f**) Co-IP experiments to detect the interaction between Cops2 and Eset. Cops2-Flag, together with GFP-V5 or Eset-V5, was overexpressed in HEK293T cells. IP experiments were carried out with Flag or V5 antibodies. (**g**,**h**) ChIP experiments to detect Eset binding (**g**) and H3K9me3 (**h**) enrichment at the *Tdpoz2*, *Sp110*, and *Ddit4l* loci. ChIP assays were performed with *GFP* and *Cops2* KD ESCs. *Tcfap2a* and *Kcnh5* are positive control regions for Eset binding and H3K9me3 enrichment, respectively. Data are shown as mean ± SD (n = 3).

We then asked whether Cops2 maintains pluripotency through mechanisms other than stabilizing Nanog protein. Microarray experiments were carried out to analyze the global expression profile changes of mouse ESCs after *Cops2* KD, as well as *Cops5* KD, as a control for disrupting the CSN. *Cops2* KD activates more genes (516) than repressed genes (139) (Fig. 4a), consistent with previously reported transcriptional corepressor function of Alien, a truncated variant of Cops2^24^,^25^. Moreover, a large fraction of Cops2 regulated genes are not affected by *Cops5* KD (Fig. 4a), further confirming the CSN-independent function of Cops2. Gene ontology analysis revealed that Cops2 regulated genes are enriched for genes involved in transcriptional regulation, embryonic morphogenesis, cell proliferation, and cell fate commitment (Fig. 4b). Thus, the transcriptional corepressor function of Cops2 might contribute to pluripotency maintenance. It is notable that genes repressed by Cops2 are enriched for 2C genes (Fig. 4c), which have been implicated in pluripotency regulation^32^. The activation of 2C genes upon *Cops2* KD, such as *Tdpoz2*, *Sp110*, and *Ddit4l*, was validated by quantitative RT-PCR, and the occupancies of Cops2 at these 2C gene loci were detected with chromatin immunoprecipitation (ChIP) (Fig. 4d,e). It has been shown that 2C genes are repressed by repressive complexes, composed of Eset, Trim28, Rif1, PCNA, Kdm1a, Hdac1/2 and Sin3a^33^,^34^, and that Alien interacts with Eset to repress *E2F1*^25^. We first confirmed the interaction between Cops2 and Eset with co-IP (Fig. 4f). Interactions of Cops2 with other repressive factors, including Rif1, Trim28, PCNA, Kdm1a, Hdac1/2 and Sin3a, were also demonstrated (Supplementary Fig. S3). Furthermore, *Cops2* KD reduces the binding of Eset and the enrichment of H3K9me3 at *Tdpoz2*, *Sp110*, and *Ddit4l* loci (Fig. 4g,h), suggesting that Cops2 facilitates the recruitment of Eset to these 2C genes.

## Discussion

In this study, we demonstrated that Cops2 itself, but not the whole CSN complex, is essential for pluripotency maintenance in mouse ESCs. First, only *Cosp2* KD, but not KD of any other CSN subunits, compromises the expression of pluripotency genes, including *Nanog* and *Oct4* (Fig. 1b). Second, *Cops2* KD results in slower proliferation rate and G2/M arrest of mouse ESCs, while KD of *Cops5* or *Cops8* does not affect ESC proliferation or cell cycle progression (Fig. 2a–c). Third, we were able to establish stable *Cops5* and *Cops8* KD ESCs. Nevertheless, attempts to construct stable *Cops2* KD ESCs were failed (Supplementary Table S1). In addition, overexpression of Cops2 promotes somatic cell reprogramming by Oct4, Sox2, Klf4 and c-Myc. Cell cycle regulation, stabilization of Nanog protein, and transcriptional repression by Cops2 might contribute to reprogramming. Yet, these mechanisms need to be tested.

Cops2 regulates the expression of *Nanog* by binding to and stabilizing Nanog protein, rather than through transcriptional regulation. Luciferase assays showed that *Cops2* KD does not change the reporter activity of the *Nanog* promoter (Supplementary Fig. S2a). Accelerated degradation of Nanog was observed upon *Cops2* KD, but not in *Cops5* and *Cops8* KD ESCs (Fig. 2a,b). *Cops2* KD reduces the expression of endogenous *Nanog*, as well as exogenous *Nanog* driven by the chicken *β-Actin* promoter (Fig. 3d,e and Supplementary Fig. S2b). In addition, the HD of Nanog is required for the down-regulation of Nanog protein induced by *Cops2* KD. By binding to the HD of Nanog, Cops2 might block the ubiquitination of lysine residues in this region, thus preventing Nanog from degradation by proteasome. Whether other mechanisms, such as post-translational modifications, are involved in regulating Nanog stability by Cops2, and whether Cops2 regulates the stability of other proteins, remain to be explored.

Alien, a truncated variant of Cops2, has been shown to be a transcriptional corepressor. We also noticed that the transcriptional corepressor function of Cops2 might contribute to pluripotency maintenance in mouse ESCs. Genes repressed by Cops2 are enriched for transcription factors, as well as regulators for embryonic morphogenesis, cell proliferation, and cell fate commitment (Fig. 4b). Moreover, 2C genes are enriched in Cops2 repressed genes (Fig. 4c). We have shown that Cops2 interacts with subunits of repressive complexes to suppress transcription (Fig. 4f and Supplementary Fig. S3). Yet, which transcription factor(s) cooperate with Cops2 in ESCs remains elusive.

Both *Cops2* and *Cops8* null blastocysts fail to give rise to ESCs^29^,^30^. Thus, *Cops2* and *Cops8*, likely the whole CSN complex, are required for pluripotency establishment in the blastocyst. How the CSN contributes to the establishment of pluripotency remains to be investigated. In addition, COPS1, COPS2, and COPS4 have been identified as OCT4 regulators in human ESCs through a genome-wide screening^26^. The discrepancy between the human ESCs data and our results from mouse ESCs might be due to species differences, as well as the naïve pluripotent status in mouse ESCs and the primed pluripotent status in human ESCs. Alternatively, false positive hits in large-scale screening might account for the seemly conflicting results. Thus, additional efforts are required to clarify the roles of COPS2 and the CSN in human ESCs.

## Methods

### Cell Culture

V6.5 mouse ESCs were cultured in growth medium consisting of 85% Dulbecco’s modified Eagle’s medium (high glucose DMEM, GIBCO), 15% fetal bovine serum (FBS, Hyclone), 2 mM L-glutamine, 5000 U/ml penicillin and streptomycin, 0.1 mM nonessential amino acids (Invitrogen), 0.1 mM 2-mercaptoethanol (Sigma), and 1000 U/ml LIF (ESGRO, Chemicon). Mouse embryonic fibroblast (MEFs) and 293T were cultured in growth medium consisting of 90% high glucose DMEM, 10% FBS, 2 mM L-glutamine, 5000 U/ml penicillin and streptomycin.

### shRNA knockdown

shRNA plasmids were constructed with the pSuper-puro system (Oligoengine) following the manufacturer’s instruction. Targeting sequences of shRNAs were the following. Cops1: 5′-AACGCACCTGATGTCAACT-3′; Cops2: 5′-GCAGTCACCAGGAACTATTCT-3′ (C2-1), 5′-GTGCATACTGGATAACACTAT-3′ (C2-2); Cops3: 5′-GTTCCTGACTTCGAAACACTA-3′; Cops4: 5′-GAACAGTTACAGATACACTAT-3′; Cops5: 5′-GGAAGCGCAGAGTATCGAT-3′; Cops6:5′-GAACCCTATGACCAAGCAC-3′; Cops7a: 5′-GGGACCTATGCGGACTACTTA-3′; Cops7b: 5′-GGAACTAGAAGACCTTATCAT-3′; Cops8: 5′-GCTCCAGAACGACATGAATAA-3′; GFP: 5′-TACAACAGCCACAACGTCTAT-3′.

### Transfection

ESCs and HEK293T cells were transfected with DNA using Lipofectamine 2000 Transfection Reagent (Invitrogen) according to the manufacturer’s protocol. For transfection into ESCs, to ensure high transfection efficiency, 1.25 μM puromycin was added into the medium 24 hours after transfection, and until cells were harvested.

### Quantitative RT-PCR

Total RNA was extracted from cells using RNeasy Mini kit (Qiagen). cDNA synthesis was performed using TransScript II First-Strand cDNA Synthesis SuperMix kit (Transgen) according to manufacturer’s instruction. PCR reactions were performed with SYBR Green Real-time PCR Master Mix (TOYOBO) in a BioRad iQ5 system. PCR cycling conditions were: 95 °C for 2 min, 40 cycles of 95 °C for 15 s, 58 °C for 15 s, and 72 °C for 30 s, and then a melting curve of the amplified DNA was acquired. Quantification of target genes was normalized with β-actin. Primer sequences were shown in Table S2.

### Western blot

Cells were lysed in lysis buffer (Beyotime), and protein concentration was measured using BCA Protein Assay Kit (Beyotime) to ensure equal loading. The samples were resolved by SDS-PAGE, followed by transferring onto a PVDF membrane (millipore). Membranes were probed with primary antibodies. Bound primary antibodies were recognized by HRP– linked secondary antibodies (GE Healthcare). Immunoreactivity was detected by ECL Plus (Beyotime) and Kodak light film. Digital images of films were taken with BioRad Molecular Imager Gel Doc XR. Primary antibodies used for Western blot are anti-Flag (sigma, F1804), anti-HA (Santa Cruz, Sc7392), Anti-V5 (Invitrogen, P/N 46-0705), anti-β-Tubulin (Huada, AbM59005-37B-PU), anti-Cops2 (Bethyl, IHC-00179), anti-Nanog (Bethyl, A300-397A), anti-Sox2 (Genetex, GTX101507), anti-Cops8 (Biomol, PW8290), anti-Rif1 (Santa Cruz, sc-65191) and anti-Oct4 (Santa Cruz, sc-5279).

### Alkaline phosphatase (AP) staining

Mouse ESCs were washed once with PBS solution, and incubated with alkaline phosphatase substrate kit III (Vector) for 20 min at room temperature.

### Colony forming assay

Mouse ESCs (200 cells per well of a 12-well plate) were cultured in ESC medium for 6–8 days. Cells were stained for alkaline phosphatase, and AP-positive colony number was counted under the microscope.

### Embryoid body differentiation

To form EBs, ESCs were cultured in 25 μL hanging drops (1000 cells/drop, ESC medium without LIF), and EBs were collected on day 4.

### Somatic cell reprogramming

To pack retroviruses, pMXs-based retroviral vectors (pMXs-Sox2, Oct4, c-Myc, Klf4, Cops2, or shRNAs targeting Cops2) were introduced into Plat-E cells using Lipofiter transfection reagent (Hanbio) according to the manufacturer’s protocol. MEFs were infected twice with different combination of viruses on day 0 and 2. After the second viral infection, the cells were cultured in ESC medium, and medium was changed every day. On day 14, the plate was stained for AP, and the number of iPSC colonies was counted under microscope.

### Luciferase reporter assay

V6.5 ESCs were seeded at a density of 1 × 10^5^ cells per well in 24-well plates, and transfected using lipofectamine 2000 (Invitrogen) with reporter plasmid (200 ng), and pRV-SV40 (8 ng, Promega), together with 600 ng control and Cops2 knockdown plasmids. At 24 h after transfection, luciferase activities were measured with the dual-luciferase reporter assay system (Promega) according to the manufacturer’s instructions. Each experiment was performed in triplicates and repeated three times.

### Co-immunoprecipitation

Cells were lysed in lysis buffer (20 mM Tris-HCl pH 8.0, 137 mM NaCl, 10% glycerol, 1% NP-40, and 2 mM EDTA) with protease inhibitor (Roche), on ice for 30 min. After centrifugation at 12,000 g for 20 min, the supernatant was collected and incubated with anti-Flag M2 magnetic beads (Sigma) or primary antibody bound protein G beads at 4 °C overnight. The beads were washed three times with lysis buffer, and the bound proteins were released from the beads by boiling the beads in 2 × SDS loading buffer for 5 min. Western blot was performed to detect the proteins present in IP samples. Primary antibodies used for co-IP are anti-HA (Santa Cruz, Sc7392) and Anti-V5 (Invitrogen, P/N 46-0705).

### Cell cycle analysis

Single-cell suspensions were prepared by trypsinization, and washed once in PBS. Cells were fixed in ice-cold 70% (vol/vol) ethanol and stored at 4 °C overnight. Following RNaseA treatment, total DNA was stained with propidium iodide (PI, Beyotime). Cells were then analyzed with a FACSCalibur flow cytometer (BD Biosciences).

### Cell apoptosis analysis

Cells were collected by trypsinization and washed with PBS. To detect cell death, an Annexin V apoptosis detection kit (Beyotime) was used. Briefly, 1 × 10^5^ cells were mixed with Annexin V FITC and PI, and incubated for 15 min at room temperature in the dark. Stained cells were analyzed with a FACSCalibur flow cytometer (BD Biosciences).

### Microarray experiment

Mouse ESCs were transfected with shGFP, shCops2 (two different shRNAs, C2-1 and C2-2), or shCops5 plasmids. Seventy-two hours after transfection, cells were harvested for RNA purification. Hybridization and scanning of the chips (Agilent-028005 SurePrint G3 Mouse GE 8 × 60K Microarray) was performed by CapitalBio Corp., Beijing, China. Differentially expressed genes were identified by comparing *Cops2* (C2-1 and C2-2) and *Cops5* KD (C5) samples to *GFP* KD (G1) sample. Genes activated or repressed in both C2-1 and C2-2 samples are considered as Cops2 repressed or activated genes, respectively. The gene ontology analysis was performed with DAVID (http://david.abcc.ncifcrf.gov/). The microarray data set (accession number GSE72536) has been deposited in the Gene Expression Omnibus database.

### ChIP assay

1 × 10^7^ ESCs were harvest and cross-linked with 1% formaldehyde. Cells were lysed and sonicated to an average size of 0.5–1 kb. For each ChIP, half of the chromatin extract was used. Anti-Flag M2 magnetic beads (Sigma) were used for Cops2-Flag ChIP. For Eset and H3K9me3 ChIP, anti-Eset (Santa Cruz, SC-66884) and anti-H3K9me3 antibodies (Abcam, ab8898), together with dynabeads protein G beads (Thermo Fisher Scientific), were used. Purified input and ChIP DNA were analyzed by real-time PCR. Relative enrichments were calculated by comparing ChIP DNA to input DNA and normalizing to the control regions (Sp110-C for Cops2-Flag ChIP, Tcfap2a for Eset ChIP, and Kcnh5 for H3k9me3 ChIP). Primers for ChIP DNA quantification were listed in Table S2.

### Statistical analysis

Data were analyzed by Student’s *t* test, unless specified. Statistically significant p values were indicated in Fig. s as follows: ***p < 0.001; **p < 0.01; *p < 0.05.

## Additional Information

**How to cite this article**: Zhang, W. *et al.* Cops2 promotes pluripotency maintenance by Stabilizing Nanog Protein and Repressing Transcription. *Sci. Rep.* **6**, 26804; doi: 10.1038/srep26804 (2016).

## Supplementary Material

Supplementary Information
