# Supplementary material for: Cops2 promotes pluripotency maintenance by Stabilizing Nanog Protein and Repressing Transcription
Source: Sci Rep. 2016 May 26;6:26804. doi: 10.1038/srep26804 (PMC4881025; doi:10.1038/srep26804)
Supplement: Supplementary Information [file srep26804-s1.pdf]

Supplementary Information

Cops2 promotes pluripotency maintenance by Stabilizing Nanog Protein and Repressing Transcription

Weiyu Zhang[1,5], Peiling Ni[1,5], Chunlin Mou[1,5], Yanqin Zhang[1,5], Hongchao Guo[1,2,5], Tong Zhao[1], Yuin-Han Loh[2,3*], Lingyi Chen[1,4*]

[1] State Key Laboratory of Medicinal Chemical Biology, Collaborative Innovation Center for Biotherapy, 2011 Collaborative Innovation Center of Tianjin for Medical Epigenetics, Tianjin Key Laboratory of Protein Sciences and College of Life Sciences, Nankai University, Tianjin 300071, China

[2] Epigenetics and Cell Fates Laboratory, A*STAR Institute of Molecular and Cell Biology, 61 Biopolis Drive Proteos, Singapore 138673, Singapore

[3] Department of Biological Sciences, National University of Singapore, Singapore

[4] State Key Laboratory of Molecular Oncology, Cancer Institute/Hospital, Chinese Academy of Medical Sciences, Beijing 100021, China

[5] Co-first authors

[*] Correspondence: yhloh@imcb.a-star.edu.sg (Y-H.L.), lingyichen@nankai.edu.cn (L.C.)

Running Title: *Role of Cops2 in pluripotency maintenance*

Supplementary Figures and Legends

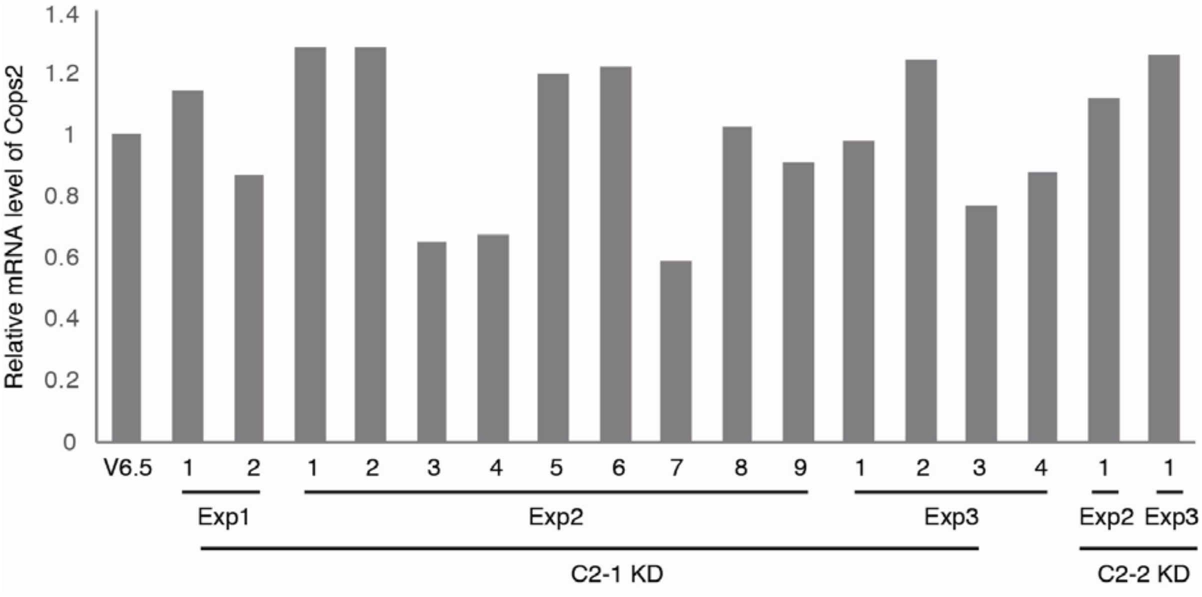

Supplementary Figure S1. The expression of *Cops2* mRNA in the surviving *Cops2* KD ESC clones shown in supplementary Table S1.

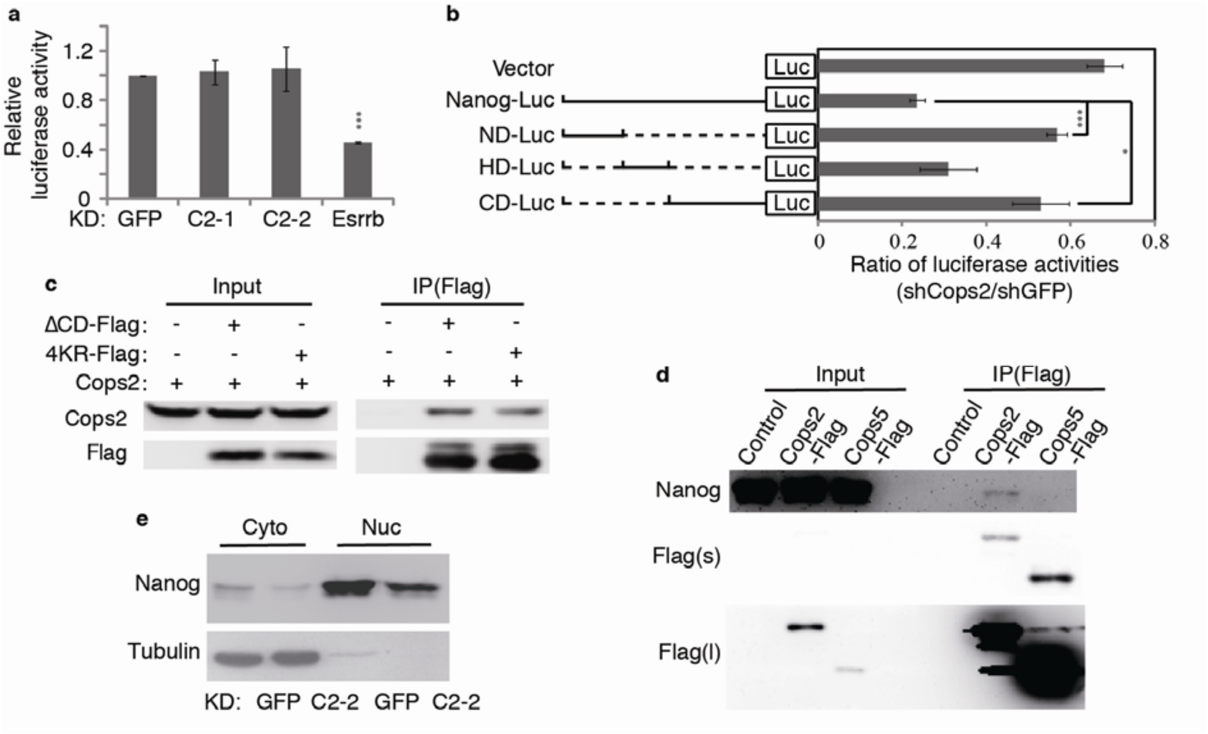

Supplementary Figure S2. Cops2 regulates the expression of Nanog by promoting Nanog protein stability, rather than transcriptional regulation. (a) A luciferase reporter vector was constructed with the 6-kb *Nanog* promoter. The *Nanog* reporter plasmid and pRV-SV40, together with shRNA plasmids targeting *GFP*, *Cops2*, or *Esrrb*, were transfected into ESCs. Twenty-four hours after transfection, luciferase activities were measured. Data are shown as mean ± SD (n=3). (b) A serial of reporter vectors were constructed by fusing full-length Nanog, ND, HD, or CD of Nanog to the luciferase, and the chicken *β-Actin* promoter was used to drive the expression of luciferases. These reporter plasmids and pRV-SV40, together with shRNA plasmids targeting *GFP* or *Cops2*, were transfected into ESCs. Twenty-four hours after transfection, luciferase activities were measured. The ratios of reporter activities in *Cops2* KD ESCs to those in *GFP* KD ESCs were plotted. Data are shown as mean ± SD (n=3). (c) The interaction between Nanog and Cops2 in ESCs. Co-IP experiments were carried out with cell extracts prepared from control ESCs, and ESCs expressing Flag-tagged Cops2 or Cops5. Short (Flag(s)) and long (Flag(l)) exposure of the anti-Flag blot are shown. (d) 4KR mutation does not affect the interaction between Cops2 and Nanog. Flag-tagged ΔCD or 4KR ΔCD Nanog mutants, together with Cops2, were overexpressed in HEK293T cells. Co-IP and Western blot were carried out to detect the interaction between Cops2 and Nanog mutants. (e) Knockdown of Cops2 does not affect the cytoplasmic and nuclear distribution of Cops2. Nuclear and cytoplasmic fractions were prepared from ESCs transfected with shRNA plasmids targeting *GFP* or *Cops2*, and subjected to Western blot assay.

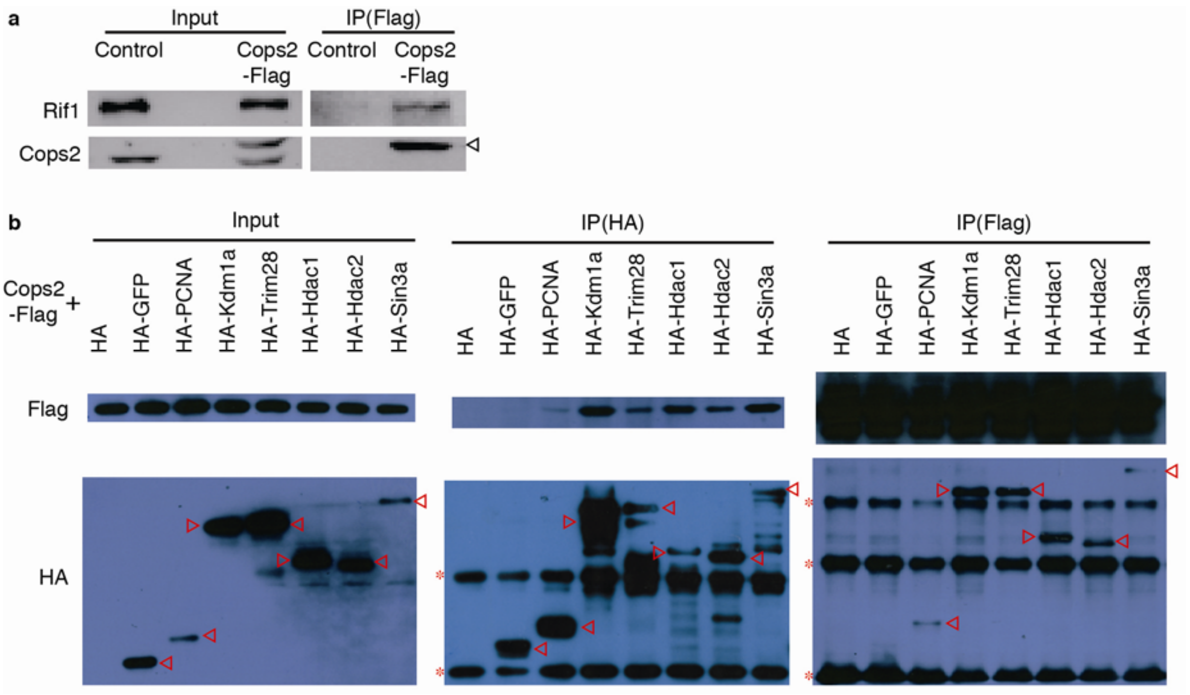

Supplementary Figure S3. Cops2 interacts with subunits of repressive complexes. (a) Co-IP experiment to detect the interaction between Cops2 and Rif1 in ESCs expressing Flag-tagged Cops2. The band of Flag-tagged Cops2 is marked with a black triangle. The lower band in the anti-Cops2 blot is the endogenous Cops2. (b) Co-IP experiments to detect the interaction of Cops2 with subunits of repressive complexes. Flag-tagged Cops2, together with HA-tagged GFP, PCNA, Kdm1a, Trim28, Hdac1, Hdac2, or Sin3a, were expressed in HEK293T cells. Anti-Flag and anti-HA Co-IP experiments were carried out with cell extracts from these cells. Specific bands in the HA blots are marked with red triangles. Non-specific and IgG bands are labelled with asterisks.

Supplementary Tables

Supplementary Table S1. Colony numbers after selection for stable shRNA knockdown ESCs

Target gene		Experiment		
		1	2	3
GFP		158	155	91
Cops1		ND	84	ND
Cops2 (*)	shRNA-1	2	9	4
	shRNA-2	0	1	1
Cops3		ND	95	89
Cops4		ND	52	ND
Cops5		ND	125	108
Cops6		ND	91	ND
Cops7a		ND	116	ND
Cops7b		ND	182	ND
Cops8		215	284	192

*: Less than 2-fold reduction of *Cops2* mRNA in all the *Cops2* KD ESC clones (supplementary Fig. S1).
ND: Not determined.

Supplementary Table S2. Primers for quantitative RT-PCR and ChIP.

Assay	Gene	Forward primer	Reverse primer
Quantitative RT-PCR	*Bmp4*	ACAGCGGTCCAGGAAGAAGAAT	TGCACAATGGCATGGTTGGT
	Cdx2	CAGTCCCTAGGAAGCCAAGTGAAA	AAGTGAAACTCCTTCTCCAGCTCC
	Flk1	CAGGAAACTACACGGTCATCCTCA	AGGAATCCATAGGCGAGATCAAGG
	Gata4	GCTATGCATCTCCTGTCACTCAGA	CCAAGTCCGAGCAGGAATTTGAAG
	Gata6	CTTCTCCTTCTACACAAGCGACCA	ATACTTGAGGTCACTGTTCTCGGG
	Hand1	AAGGATGCACAAGCAGGTGAC	TTTAATCCTCTTCTCGCCGGG
	Nanog	TACAAGGGTCTGCTACTGAGATGC	TTGGGACTGGTAGAAGAATCAGGG
	Nestin	CTGGATCTGGAAGTCAACAGAGGT	ATCCTCAGTTTCCACTCCTGTAGC
	Nkx2.5	ACTATGCCCTGTCCCTCAGATTTC	TCCTAGTGTGGAATCCGTCGAAAG
	Oct4	ATCAGCTTGGGCTAGAGAAGGATG	AAAGGTGTCCCTGTAGCCTCATAC
	Pax6	TAACGGAGAAGACTCGGATGAAGC	GGGCAAACACATCTGGATAATGGG
	Sox17	CCCAACACTCCTCCCAAAGTATCT	TCTCTGTCTTCCCTGTCTTGGTTG
	Sox2	GCGGAGTGGAAACTTTTGTCC	CGGGAAGCGTGTACTTATCCTT
	T	CATCGGAACAGCTCTCCAACCTAT	TACCATTGCTCACAGACCAGAGAC
	β-Actin	CAGAAGGAGATTACTGCTCTGGCT	TACTCCTGCTTGCTGATCCACATC
	Ddit4l	CCTGGGAGTCTGCTAAGTG	TTTGGTTTGCTTTGATCTGGAC
	Sp110	AAAGCCCCGTCAAGATGAG	ATCCTTGTTCTCTGTGTCTTGG
	Tdpoz2	AAAGCCCAGTGTTCTCCTTAG	TGCCCTAACTTGTCTCTTTGG
	Cops1	GTATGAAGAGATACACCGGAAGC	TCCAGTTTCAACAGAGCCTTC
	Cops2	CATCCCTCACCCACTAATCATG	TCTTGGGCTTCCTGATTCATC
	Cops3	AGAAAACAGCCCCTTCGAG	AGAAAACAGCCCCTTCGAG
	Cops4	CGCCCAGAGGTACAATGAG	AAAAGAGTAGCCAGCATCCG
	Cops5	CGCCCAGAGGTACAATGAG	AAAAGAGTAGCCAGCATCCG
	Cops6	TGAACCCTATGACCAAGCAC	GATCCGTTCAGCTTCCTCAG
	Cops7a	TGTGTATGCTGATGTCCTTCG	AACACAACCTCACAGCCCAC
	Cops7b	GGAAGTGGATTTCTGCATTGG	TCTCTTTGTACTGGTTGGCTC
	Cops8	ACTTGCTCCAGAACGACATG	GATGGTTGTATAGATCCCAGGG
ChIP	*Sp110*	TGAGGAGACATTCGGCATCTA	GTCCTCTCACTCTCAGTCCAA
	Ddit4l	CTGTCGGTTACTGCAGGTATATG	TTCCACCGGGACAAAGTTATAG
	Tdpoz2	AGCTAGACAGATATGACCAACAC	CAGGCAGAAAGTAAGGCTACA
	Cops2	CAGCTCCTTGAACTGGATCAT	ACAGCCTGGTTCAGAGAATTTA
	Tcfap2a	CCTGTGGCCGCAAGGATGACTGAGT	GCACTTTGCGCTAACCCAGAGAGTAGCTCC
	Kcnh5	AGGTCCTACAGTGTTTGTGTATC	GAGCTGGAATATGGCGAGAA
	Sp110-C	GTCTCATGCTTACTTCTTGCAC	ACTGTGGTACACGCTCAC
